# The User-Centered Design of a Clinical Dashboard and Patient-Facing App for Gestational Diabetes

**DOI:** 10.1177/19322968241301792

**Published:** 2024-11-29

**Authors:** Jasmine R. Kirkwood, Jane Dickson, Marryat Stevens, Areti Manataki, Robert S. Lindsay, Deborah J. Wake, Rebecca M. Reynolds

**Affiliations:** 1 Centre for Cardiovascular Science, Queen’s Medical Research Institute, The University of Edinburgh, Edinburgh, UK; 2 Medical School, University of Dundee, Dundee, UK; 3 MyWay Digital Health, Dundee, UK; 4 School of Computer Science, University of St. Andrews, St. Andrews, UK; 5 School of Cardiovascular and Metabolic Health, The University of Glasgow, Edinburgh, UK; 6 Usher Institute, The University of Edinburgh, Edinburgh, UK

**Keywords:** digital health, gestational diabetes mellitus, health education, mHealth, user-centered design

## Abstract

**Background:**

The number of pregnancies affected by gestational diabetes mellitus (GDM) is growing. With the increased use of smartphones and predictive modeling, a mobile health (mHealth) solution could be developed to improve care and management of GDM while streamlining care through risk stratification.

**Methods:**

A user-centered mHealth tool was designed from ethnographic observations and 11 semi-structured interviews (six health care professionals [HCPs] and five women with GDM), followed by iterative changes and evaluation from three feedback groups with 31 participants (17 HCPs, 14 researchers) and 13 questionnaires with women with GDM.

**Results:**

“MyGDM” includes a clinical dashboard that centralizes the clinic’s patients, highlighting off-target blood glucose and predicting the need for pharmacological intervention. It is linked with a patient-facing app that includes structured education, culturally inclusive language options, and meal ideas. Through the feedback sessions, iterative changes were made around visualization and patient safety, and participants were positive toward the potential user experience. In the 13 questionnaires with women with GDM, 100% said it would fit into their lifestyle and help them manage GDM. Educational resources and the “request a call” functions were well received with 61.5% (8/13) and 69.2% (9/13) saying they were very likely or likely to use these, respectively.

**Conclusion:**

A user-centered mHealth tool consisting of a clinical dashboard linked with a patient-facing app for GDM care and management has been designed. Evaluation of the interactive design by end users was positive and showed that it met their needs.

## Introduction

Gestational diabetes mellitus (GDM) is hyperglycemia that is first recognized during pregnancy.^1-3^ Gestational diabetes mellitus is one of the most common pregnancy complications,^4^ affecting approximately 5.8% of pregnancies in Europe^5^ and up to 30% globally.^6^ It is expected to increase due to the rise in obesity^7,8^ and maternal age.^8^ Women who had GDM have a ten-fold increase in developing type 2 diabetes,^9^ and a two-fold increase in developing early cardiovascular disease,^10,11^ with their offspring at a greater risk of childhood obesity and diabetes.^2,3,12^

There is a short period between diagnosis of GDM and birth where a woman with GDM must comprehend the consequences of the diagnosis, while managing the additional burden it places on their pregnancy. This places considerable stress on the women during their pregnancy.^13-15^ In addition, care is time consuming, with women with GDM spending over two hours attending a multidisciplinary antenatal diabetes clinic, every one to two weeks, excluding travel time.^16^ As such, remote monitoring and remote consultation could potentially be beneficial. Clinical needs are diverse and therefore there is the potential to use risk stratification to identify those likely to need pharmacological therapy to streamline care to those most in need of regular face-to-face clinic review.^17^

Mobile health (mHealth) is any service provided through a mobile device within a health care setting. Mobile health could be a good addition to standard care for GDM,^18^ as it allows patients to be more involved with their treatment and is not restricted to a time or place.^19^ The use of smartphone apps and online dashboards for GDM remote monitoring has been found to increase women’s satisfaction, confidence, and knowledge regarding GDM, and to optimize clinical time without worsening glycemic control.^20^ Such tools could lead to personalization of care with the potential for rapid integration into the clinic, as smartphone use is high among pregnant women with diabetes.^16^

Currently, most mHealth apps for GDM mainly report patient information, alerts around medication and appointments, or education^21^; however, there is a lack of GDM-specific information or courses,^22^ and a limited number including artificial intelligence or machine learning.^21^ Furthermore, to our knowledge, none of the clinically used apps have been designed with their end users.^22^ Hence, there is a gap, to develop with end users, an mHealth tool for GDM care and management, to help women to self-manage their GDM, and with the potential to personalize care through pharmacological therapy risk stratification.

We aimed to conduct a user-centered design and an evaluation of a potential mHealth tool to facilitate the management of GDM.

## Methods

**Figure 1.**
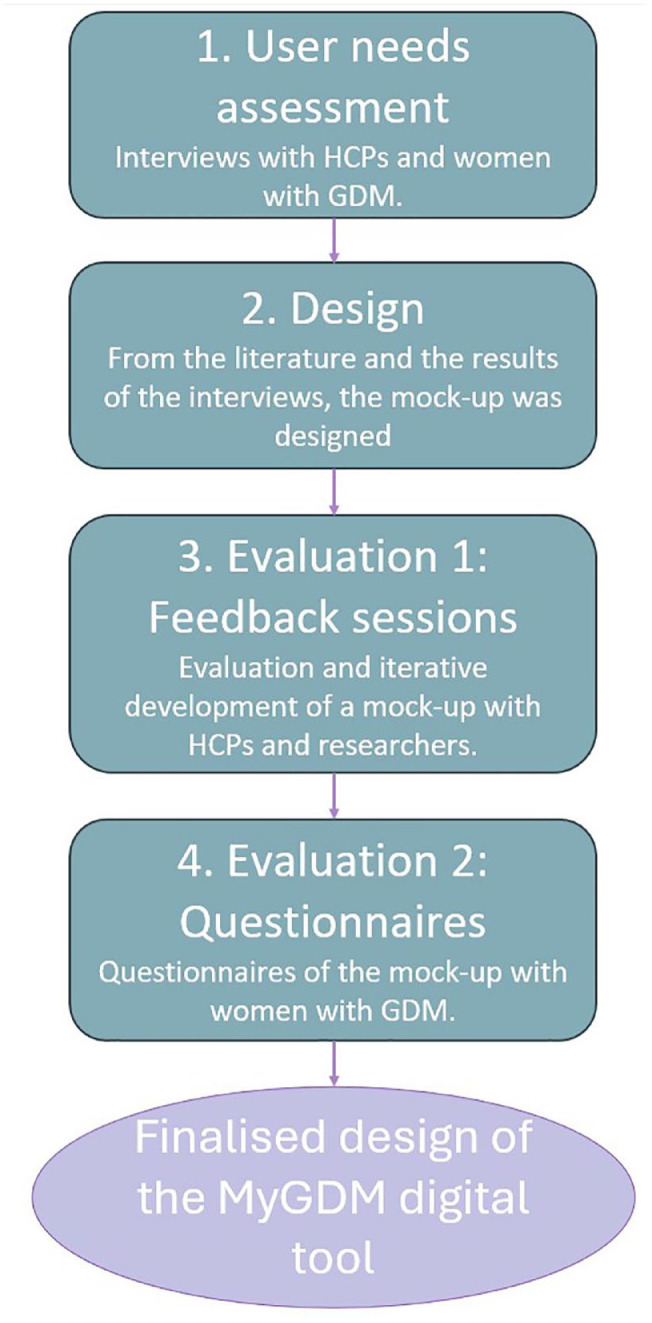
Study design schematic. Abbreviations: HCPs, health care professionals; GDM, gestational diabetes mellitus.

We used a user-centered approach,^23^ in which we included the end users (health care professionals [HCPs] and women with GDM) in the design and evaluation of a new mHealth tool. The interactive design mock-up is a clinical dashboard linked to a patient-facing app that was developed through four stages, as shown in Figure 1.

### User Needs Assessment

The aim of the user needs assessment was to understand the views, options, and acceptance of digital technologies within GDM care and management, and what end users would want from such technologies. The results then informed the initial design. To familiarize with the setting, first, four antenatal diabetes clinics in three hospitals in Edinburgh and Glasgow were observed between September and December 2022. This was followed by 11 (HCP *n* = 6, women with GDM *n* = 5) semi-structured interviews that took place between January and July 2023 (topic guide in Supplemental Material 1). Semi-structured interviews allow for an in-depth exploration of the research topics and a greater understanding of the users’ wants,^24^ hence a minimum of six to ten participants are deemed sufficient.^25^

Participants were recruited from flyers and e-mails in the antenatal diabetes clinics in Edinburgh and Glasgow. Written and verbal consent was obtained before interviews which were transcribed by a third-party, first-class secretarial services (https://www.1stclass.uk.com/) or a researcher. Transcripts were coded using NVivo (12 software V3). Thematic analysis of transcripts was carried out using the six-step framework described by Braun and Clarke^26^: (1) familiarization with the data, by reading through transcripts; (2) coding the transcripts with 10% double coded (JRK and JD); (3) searching for themes by reviewing the codes and summarizing the interviews; (4) reviewing themes; and (5) defining and (6) naming themes. Codes and themes were refined through discussions throughout.

### Design

From the literature^27-35^ and the results of the user needs assessment, an interactive design, consisting of a clinical dashboard for HCPs linked with a patient-facing app, was developed on Figma (www.figma.com/prototyping/). The aim was to design a tool that met our end users’ needs and desires.

### Evaluation

The mHealth tool was evaluated through feedback sessions with HCPs and researchers and questionnaires with GDM women. The aim was to appraise and identify areas of improvement with end users.

### Feedback Sessions

Three feedback sessions of 31 participants (17 HCPs and 14 researchers) from Edinburgh and Glasgow were recruited through the researcher team’s network (RMR and RSL). Feedback sessions took place between January and February 2024 and were either hybrid or online with a mean (standard deviation) duration of 32 (3.9) minutes. Verbal consent was gained before each session. During the session, the clinical dashboard and patient-facing app were demonstrated, followed by an open discussion (topic guide in Supplemental Material 2). After each session, the automatic Teams transcripts were reviewed against the video, alongside notes taken. Iterative changes to the interactive design mock-up were made and then presented to the next feedback session. After the final session, transcripts were thematically analyzed, again using the Braun and Clarke framework.^26^

### Questionnaires

Women with GDM were recruited between February and April 2024, at antenatal diabetes clinics in Edinburgh (*n* = 12, 92.3%) or if they had been previously interviewed and agreed to participate in the questionnaire stage (*n* = 1, 7.7%). Data saturation was reached after 13 questionnaires, where no more themes emerged. Verbal consent was gained, and then participants were shown a short video (2:48 minutes) demonstrating the patient-facing app and asked to complete a questionnaire (Online Surveys, https://www.onlinesurveys.ac.uk/; Supplemental Material 3). The questionnaire enquired about the app’s potential usefulness, options on features, and areas of improvements. A short questionnaire was chosen to reduce the burden on participants. Themes of free-text questions were identified by reading through answers and grouping into similar topics and then naming themes. Basic descriptive statistics were completed on Microsoft Excel (Version 2405).

### Participant Inclusion Criteria

Participants were included if they were (1) ≥16 years old, (2) had given consent to take part in the study, (3) able to read and write, and (4) willing to use their e-mail address for correspondence. Women with GDM (1) had GDM/previous GDM and were treated in Edinburgh or Glasgow within the last two years, and (2) were not pre-diabetic or had pre-gestational diabetes. Healthcare professionals had (1) worked in GDM care in Edinburgh/Glasgow in the last two years, and (2) at least one year of experience within GDM care. Participants in the feedback sessions had worked or researched pregnancy care in Edinburgh or Glasgow in the last two years and had an understanding of GDM.

### Ethics

The study received ethical approval from the Research Ethics Committee (reference number 22/NW/0164). The study was co-sponsored by NHS Lothian and the University of Edinburgh through the Academic and Clinical Central Office for Research and Development.

## Results

### User Needs Assessment

The characteristics of the 11 participants who were interviewed for the user needs assessment are described in Table 1.

**Table 1.** Characteristics of Participants in the User Needs Assessment.

Health care professionals (*N* = 6)		
Variable	Value	Number (percentage response)
Location	Edinburgh Glasgow	4 (66.7%) 2 (33.3%)
Role	Consultant endocrinologist/physician Consultant obstetrician Dietitian Diabetes specialist nurse	3 (50.0%) 1 (16.7%) 1 (16.7%) 1 (16.7%)
Women with gestational diabetes (*N* = 5)		
Variable	Value	Number (percentage response)
Location	Edinburgh	5 (100.0%)
Age (years)	21-25 31-35 36-40	1 (20.0%) 1 (20.0%) 3 (60.0%)
Ethnicity	White Mixed Black/African/Caribbean/Black British	2 (40.0%) 2 (40.0%) 1 (20.0%)
Gestational diabetes diagnosis before the interview	8-14 days ago 3-6 months ago 7-12 months ago	1 (20.0%) 3 (60.0%) 1 (20.0%)
Gestational diabetes in a previous pregnancy	Yes No, first pregnancy	4 (80.0%) 1 (20.0%)
Treatment for gestational diabetes at the time of the interview	Metformin Insulin Metformin and insulin	3 (60.0%) 1 (20.0%) 1 (20.0%)

Four themes with 13 subthemes emerged from the interviews, shown in Table 2 alongside illustrative quotes. Participants expressed a desire for greater availability of information on GDM and diet, including the need for it to be culturally inclusive and credible. Additionally, a need to improve the identification of issues through highlighting and notification was expressed, with the potential to streamline care through risk prediction. However, concerns over the safety of predictions were also raised. Finally, time constraint was a main barrier; it is time consuming for HCPs to manage the growing number of women with GDM, while women with GDM are required to fit in and attend additional appointments on top of their other commitments.

**Table 2.** Themes, Subthemes, and Illustrative Quotes From the Semi-structured Interviews With Health Care Professionals and Women With Gestational Diabetes.

Theme Frequency (percentage)	Subtheme Frequency (percentage)	Illustrative quote
Time constraints Overall: 10 (90.9%) HCPs: 6 (100.0%) Women with GDM: 4 (60.0%)	Time management and workload of HCPs Overall: 4 (36.4%) HCPs: 4 (66.7%) Women with GDM: 0	“Something that could be done in a completely different way and free up time to allow us to work more efficiently and utilize the time in a different way.” (HCP 3)
	Patients’ parental, work, and life commitments Overall: 8 (72.7%) HCPs: 4 (66.7%) Women with GDM: 4 (80.0%)	“I’ve no time. Most of the time I have just enough time in my brain to remember to do my blood sugar, and then it’s back into the kids.” (GDM Woman 2)
Barriers to current GDM care Overall: 9 (81.8%) HCPs: 6 (100.0%) Women with GDM: 3 (60.0%)	Technical issues Overall: 7 (63.6%) HCPs: 5 (83.3%) Women with GDM: 2 (40.0%)	“(blood glucose monitor) sometimes there’s IT issues. So not everybody shares their data.” (HCP 6)
	Appointment attendance Overall: 7 (63.6%) HCPs: 4 (66.7%) Women with GDM: 3 (60.0%)	“With my job, it’s difficult to take time off in general. So, it’s quite difficult when they’re like, this one is going to be on a computer, and you’re like, well, I need to get cover, [. . .] It’s just an added in extra.” (GDM Woman 1) “We get lots of DNAs (did not attend) for people that just go like, well, I am just going to ignore this.” (HCP 4)
	Information, resources, and education Overall: 4 (36.4%) HCPs: 2 (33.3%) Women with GDM: 2 (40.0%)	“The problem is that at booking so much information has to be given out at booking and they (woman with GDM) are bombarded.” (HCP 4)
	Cultural, social, and language Overall: 6 (54.5%) HCPs: 6 (100.0%) Women with GDM: 0	“There may be cultural barriers as well in terms of what they’re happy to do and understanding sharing of information.” (HCP 1)
Suggestions for digital tool Overall: 11 (100%) HCPs: 6 (100%) Women with GDM: 5 (100%)	Highlighting off-target blood glucose readings Overall: 5 (45.5%) HCPs: 3 (50.0%) Women with GDM: 2 (40.0%)	“Dashboard and then you just see who needs to be contacted, that would be amazing.” (HCP 5)
	Notification and prompts Overall: 9 (81.8%) HCPs: 6 (100.0%) Women with GDM: 3 (60.0%)	“Kind of prompt or troubleshoot it quickly, and be like, try this. Because as soon as I put in evening snack, right away it helped.” (GDM Woman 1)
	Predictions and risk stratification Overall: 8 (72.7%) HCPs: 6 (100.0%) Women with GDM: 2 (40.0%)	“I’m sure you could be predicting that (medication) a bit earlier. It’s not that we don’t get there, we do, but we could probably be narrowing the time and improving the delivery of care in that way.” (HCP 2)
	Diet management Overall: 5 (45.5%) HCPs: 2 (33.3%) Women with GDM: 3 (60.0%)	“Recipes. [. . .] Some little quick and easy, like, here’s something that you can change in your cooking. Take that recipe that you love and if you just do this.” (GDM Woman 1)
	Information and education Overall: 5 (45.5%) HCPs: 4 (66.7%) Women with GDM: 1 (20.0%)	“It would be really good to have lots of really good information all in the one place.” (HCP 4)
Safety and trust in data-driven solutions Overall: 4 (36.4%) HCPs: 4 (66.7%) Women with GDM: 0	Information produced by digital tools Overall: 3 (27.3%) HCPs: 3 (50.0%) Women with GDM: 0	“A process where you were very confident that the information going back to the patient was accurate and appropriate.” (HCP 2)
	Predictions made by digital tools Overall: 3 (27.3%) HCPs: 3 (50.0%) Women with GDM: 0	“Any predictive tool should be in conjunction to clinical care and that really the last signoff should be with the doctors to oversee whether or not the algorithms are making sense almost to each individual patient.” (HCP 1)

Quotes have been identified with participant type and number, and nonrelevant sections have been denoted [. . .].

Abbreviations: HCPs, health care professional; GDM, gestational diabetes mellitus.

The results of the interviews were then used to develop features for the initial design, in accordance with available literature.^27-35^

### Feedback Sessions

There were three feedback sessions, with a total of 31 participants (17 HCPs and 14 researchers). After each feedback session, iterative changes were made and presented to the next group (feedback session participant characteristics and iterative changes, Supplemental Material 2).

Clinical adaptation and the practicality of using the digital tool were discussed. This included the ability to set up clinic-wise self-monitoring blood glucose (SMBG) targets for all women with GDM, whether these readings would be taken pre-prandial or post-prandial, and the option to remove features that do not suit the clinic’s practices. Further, there were discussions about who would be responsible for checking the dashboard and how a patient’s request for a call would be notified. For example, there was the suggestion to have an e-mail notification or to be able to filter the dashboard for call requests. Patient safety is key and was raised in many different ways throughout the feedback sessions. An indicator was added to identify if SMBG had been edited or added manually by patients; this was to allow transparency around SMBG and to ensure that clinical decisions could be made accurately. Additionally, there were concerns over misinformation spreading easily in the “GDM Community” forum, which was incorporated following recommendations from literature,^27^ as it would be unregulated. Visualization of the patient’s SMBG through graphs and logbook formatting to simplify reviewing was discussed. All feedback sessions were positive regarding the aesthetics and potential user experience.

### Questionnaires

In total, 13 women with GDM completed the questionnaires, most were 31 to 40 years old, white, diagnosed seven to nine months ago, had children, did not have previous GDM, and were being treated using diet or metformin (characteristics of questionnaire participants, Supplemental Material 3).

The responses to the quantitative questions are shown in Table 3. All the participants thought that the patient-facing app would fit into their lifestyle and help them manage their GDM. Using a 5-point Likert scale from very likely to very unlikely, 69.2% (9/13) and 61.5% (8/13) of participants indicated that they were likely or very likely to use the “request a call” function and educational materials, respectively, while the eCourse had a more varied response.

**Table 3.** Quantitative Questionnaire Responses Evaluating the Patient-Facing App.

Question	Response	Number (percentage response)
Do you think the app presented would have helped you manage your gestational diabetes?	Yes	13 (100)
	No	0
Could you see the app fitting in with your lifestyle to help manage your gestational diabetes?	Yes	13 (100)
	No	0
On a scale of 1 to 5, 1 being very unlikely and 5 being very likely, how likely, if you needed to, would you have used the “request a call” feature?	Very likely (5)	5 (38.5)
	4	4 (30.8)
	3	3 (23.1)
	2	1 (7.7)
	Very unlikely (1)	0
On a scale of 1 to 5, 1 being very unlikely and 5 being very likely, how likely would you have used the “Know more” educational resources?	Very likely (5)	5 (38.5)
	4	3 (23.1)
	3	4 (30.8)
	2	1 (7.7)
	Very unlikely (1)	0
On a scale of 1 to 5, 1 being very unlikely and 5 being very likely, how likely would you have used the eCourse/quiz?	Very likely (5)	3 (25)
	4	4 (33.3)
	3	3 (25)
	2	2 (16.7)
	Very unlikely (1)	0

There were five free-text questions (Supplemental Material 3), in which four themes with eight subthemes emerged (Table 4). Participants expressed how helpful meals and exercise ideas would be for managing their GDM, as these were areas that they struggled with. There was appreciation for the comprehensive and accessible information, and this was also shown in the Likert responses. Easy access to SMBG readings with visualizations was seen as beneficial, especially with the ability to manually add or edit readings which could compensate for technical issues. Participants particularly valued the ability to contact their health care team through “request a call” and that the information was tailored to GDM, all of which would help them self-manage their GDM. When asked what they liked least or was missing from the patient-facing app, participants responded with nothing and that it had everything they wanted.

**Table 4.** Themes, Subthemes, and Illustrated Quotes From the Free-Text Responses in the Questionnaire on the Patient-Facing App.

Theme	Sub-theme	Illustrative quote
Managing lifestyle changes	Diet	“Suggestion for meal prep. As I struggle with it every day.” P2 Q3
	Exercise	“Meal ideas would have been very helpful and exercise too!” P1 Q1
Facilitating self-management	Education resources	“It was full of information that is useful.” P6 Q3 “Helpful information is great.” P13 Q3
	Blood glucose tracking	“Looks easy to understand and find your readings.” P12 Q3
	General gestational diabetes self-management	“My glucose levels have been up and down and a request for a call button would help with the anxiety of calling someone.” P3 Q2
Contact and communication with the clinical team	—	“Ease of getting in touch with hospital team/receiving messages from them.” P9 Q3
General app usability	User experience and interface	“Good layout and lots of information.” P6 Q8
	Useful and helpfulness	“It’s easy to navigate and has everything I need.” P2 Q3
	Tailored to gestational diabetes	“Relevant to my current situation, I feel [current blood glucose monitoring app] isn’t aimed at me.” P8 Q3

Quotes have been identified by participant number (P), along with the questions Q1: Do you think the app presented would have helped you manage your gestational diabetes? [Comments], Q2: How likely, if you needed to, would you have used the “request a call” feature? [Comments], and Q3: What did you like most about the app?

### Final Design

The mHealth tool, “MyGDM,” was designed from results of the interviews and refined through feedback sessions and questionnaire. A description of the key features of the final design alongside the rational can be seen in Table 5.

**Table 5.** mHealth Tool Key Features With Rationale and Sources.

mHealth tool key features	Rational and source
Clinical dashboard homepage 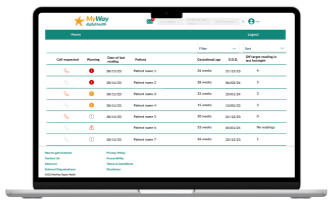	A major issue that arose during the clinical observation and interviews is that HCPs must search each patient’s name to review their SMBG readings. There was a clear need for a dashboard that held all patients currently in the clinic centrally. Furthermore, highlighting off-target SMBG was a theme from the interviews, and adapting a simple traffic light system to achieve this was implemented on the clinical dashboard homepage. The addition of filtering and sorting came from feedback sessions 2 and 3.
Medication risk prediction 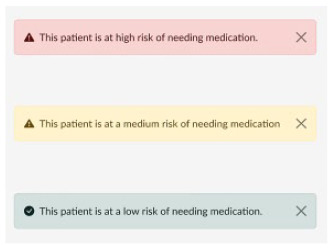	During the interviews, HCPs spoke of an increase in the GDM population, and that care is time intensive for both HCPs and patients. Therefore, streamlining care by predicting high-risk patients would allow for rapid treatment escalation and could reduce the burden on the care system. In addition, during feedback session 2 it was said that it could help identify which patients would need closer follow-up.
Viewing a patient’s page 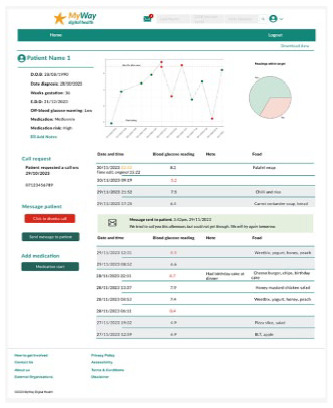	The patient’s page is a solution to include all the patients’ inputs. The addition of visualization and logbook formatting came from feedback sessions 1 and 2. The ability for a woman with GDM to manually add or edit their SMBG was included because of the study by Safiee et al.^27^ It was then in feedback session 3 that highlighting those readings that had been edited was added.
Communication (Request a call, and messages from HCPs) 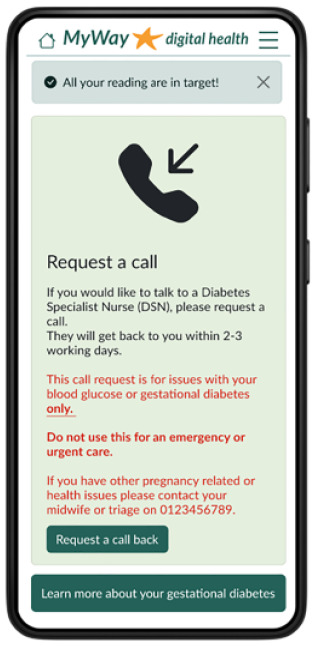	To increase app engagement,^28^ and to make contacting HCPs for support more convenient,^29^ “request a call” function and messages from HCPs were included in the digital tool. From the questionnaires, the ability to request a call was well received.
App homepage 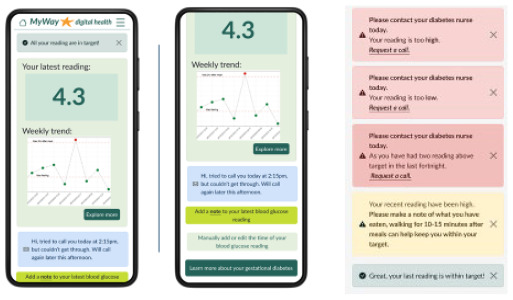	The key information needed to self-manage GDM is displayed on the homepage. This aims to be as efficient as possible and to fit into women with GDM’s lifestyle which was a theme that emerged from the interviews. Additionally, women with GDM wished to have all their data summaries onto one page.^36^ Visualization and messages of SMBG reading using a simple traffic-light system allow for real-time feedback. The message about the latest readings has been adapted from Blair et al’s study.^30^
Education: Know more and eCourse 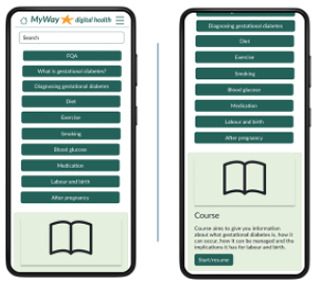	During the interviews, there was a clear voice for more information and education on GDM from both women with GDM and HCPs, which was consolidated in the questionnaires. In addition, it has been found that education can improve quality of life and self-efficacy.^31^ Structured smartphone-based education for GDM is a low-cost way to improve self-efficacy.^32^ But reliable information on GDM is hard to acquire,^27^ which was also emphasized in our interviews with HCPs. Therefore, to include reliable information, we included educational articles.
Food diary and log 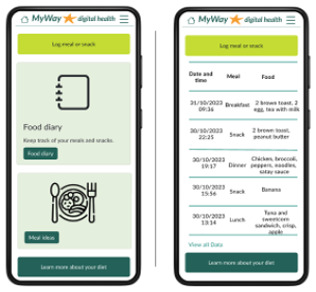	A food log and diary to help facilitate women to self-manage GDM. Tracking meals could help women identify which foods spike their blood glucose that should then be avoided. In addition, it has been expressed that participant interviewed for a theatrical pregnancy app wanted to monitor diet.^33^
Meal ideas 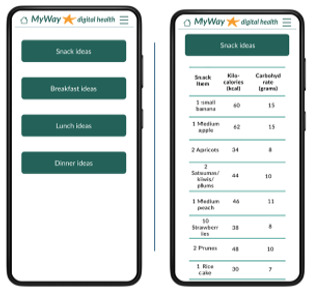	During the interviews with women with GDM, meal and recipe ideas were regularly discussed as desirable. During the questionnaires, the inclusion of meal ideas was reported as beneficial. Culturally inclusive meal options were included.

The finalized interface design consists of a clinical dashboard linked with a patient-facing app. It is intended that SMBG readings will be uploaded from the women’s blood glucose meter to their app via Bluetooth, and once connected to the Internet, readings are then shared with the clinic.

At the clinical setup, clinical glycemic targets are set, and features can be removed. Patients would be sent a clinic code to download and register on the app before their appointment.

The clinical dashboard’s homepage has all the women with GDM currently in the clinic, with prompts regarding if any women have requested a call and a simple traffic-light system showing if a woman has uploaded their SMBG readings and how in-target they are. The dashboard can also be filtered and sorted by location, medication, call requests, and warnings. The patient page has visualization, including a scatter graph of SMBG and a pie chart of in-target SMBG, to simplify the identification of trends in women’s SMBG and a logbook to aid with reviewing and making clinical decisions. Basic patient information including medication is displayed in a sidebar.

Additionally, there is a function at booking, to predict if a woman would potentially need pharmacological intervention. The clinic could use this information to streamline care, by identifying high-risk patients who would need more clinical time compared to low-risk patients who may be able to manage through diet therapy and remote monitoring.

The patient-facing app for women with GDM contains visualization and notices regarding their SMBG, reminders of targets and medication, and the ability to add notes, log food/meals, or manually add or edit SMBG. Educational articles include videos and text on GDM topics, an eCourse, and meal ideas. Language options, at app setup, were included because of the diverse GDM population. Finally, there is an ability to request a call and view any messages from the clinical team.

A GDM community forum which was initially included from the results of Safiee et al^27^ was removed from the final design because concerns over patient safety with the potential to spread misinformation were raised during the feedback sessions.

Furthermore, during the interviews and feedback sessions it was considered how the app could be used to improve attendance of postpartum HbA1c, so education articles and a postpartum reminder e-mail/notification were added.

## Discussion

We designed an interactive interface of a user-centered mHealth tool consisting of a clinical dashboard linked to a patient-facing app for GDM care and management, through key stakeholder interviews and a literature review. This was evaluated positively through feedback sessions and questionnaires.

Key features of the mHealth tool included a centralized clinical dashboard; promoting and highlighting off-target SMBG; credible education; meal ideas; data visualization; facilitating communication; and predictive risk stratification.

A time-consuming task that was highlighted by HCPs was the current method of inputting each patient to review SMBG. To combat this, a centralized clinical dashboard using a traffic light system to indicate priority patients was developed. This allows for remote monitoring which is time efficient for both clinics^37,38^ and women with GDM^16,39^ and reduces the need for in-person appointments.^40^ From the questionnaires, the ability to request a call was well received by women with GDM, with 69.4% likely to use it. In the feedback session, HCPs could see that this would help women manage themselves better but were concerned about who would monitor the requests. Remote monitoring in combination with “request a call” could reduce the number of appointments and therefore fit better into a GDM woman’s lifestyle by reducing the burden of traveling and attending appointments, which can often be a barrier.^27,29^,^38-40^

Women with GDM in the interviews requested education and meal ideas, and so these were included and then well received with 61.5% of women completing the questionnaires indicating that they would be likely to use them. Education can improve the quality of life and self-efficacy for women with GDM,^31^ especially as women with GDM use the Internet as a supplementary resource for information,^41^ suggesting that there is a need for credible information sources. Hence, evidence-based GDM-specific education resources were developed and included. Structured smartphone-based education for GDM has been shown to improve self-efficacy^32^ and so an eCourse was included. In addition, language options and culturally inclusive meal ideas were included to reduce cultural and language barriers that have been reported.^42,43^

The clinical dashboard could identify patients’ risk of need for medication which could streamline care through risk stratification. Models have been developed to predict pharmacological intervention,^44-56^ but to our knowledge, none have been included within a digital tool or implemented clinically. The use of predictive modeling in GDM could be a useful adjunct as a clinical decision support tool for HCPs.

A review of 11 mHealth apps for GDM that use artificial intelligence^21^ found only one app (GDmHealth) that has been clinically implemented in England.^57^ GDmHealth^58^ is GDM specific and helps with the management of SMBG, and enables two-way communication between HCPs and patients but does not provide any education. A scoping review including 30 studies on the use of mHealth for GDM found that half (*n* = 14) were for SMBG, and other common features included real-time feedback, communication with professionals, and education,^59^ all of which we have included in mHealth tool, in addition to developing with our end users.

Diabetes technology is diversifying,^60,61^ and as such, the mHealth tool will be adaptable for the inclusion of new technologies for GDM. Future work includes development of predictive models for pharmacological intervention, in conjunction with back-end building and integration into MyWay Clinical and obtaining necessary regulatory standards for medical devices.

### Strengths and Limitations

One strength is the user-centered design and evaluation used. This prioritizes needs and wishes of both women with GDM and HCPs and produces a more usable and effective digital tool. However, there was a lack of ethnic diversity and with self-selecting sampling increases the bias toward those who were more engaged with their care and, hence, may not be representative of the whole GDM population.

## Conclusion

We have designed a user-centered mHealth tool consisting of a clinical dashboard and a patient app for GDM care and management. Evaluation of the interactive design by end users was positive and showed that it met their needs. Future work includes the development of predictive modeling and back-end, and then piloting to ensure that it can function and improve outcomes within a clinical setting.

## Supplemental Material

sj-docx-1-dst-10.1177_19322968241301792 – Supplemental material for The User-Centered Design of a Clinical Dashboard and Patient-Facing App for Gestational Diabetes Supplemental material, sj-docx-1-dst-10.1177_19322968241301792 for The User-Centered Design of a Clinical Dashboard and Patient-Facing App for Gestational Diabetes by Jasmine R. Kirkwood, Jane Dickson, Marryat Stevens, Areti Manataki, Robert S. Lindsay, Deborah J. Wake, Dip (Med Ed) and Rebecca M. Reynolds in Journal of Diabetes Science and Technology

sj-docx-2-dst-10.1177_19322968241301792 – Supplemental material for The User-Centered Design of a Clinical Dashboard and Patient-Facing App for Gestational Diabetes Supplemental material, sj-docx-2-dst-10.1177_19322968241301792 for The User-Centered Design of a Clinical Dashboard and Patient-Facing App for Gestational Diabetes by Jasmine R. Kirkwood, Jane Dickson, Marryat Stevens, Areti Manataki, Robert S. Lindsay, Deborah J. Wake, Dip (Med Ed) and Rebecca M. Reynolds in Journal of Diabetes Science and Technology

sj-docx-3-dst-10.1177_19322968241301792 – Supplemental material for The User-Centered Design of a Clinical Dashboard and Patient-Facing App for Gestational Diabetes Supplemental material, sj-docx-3-dst-10.1177_19322968241301792 for The User-Centered Design of a Clinical Dashboard and Patient-Facing App for Gestational Diabetes by Jasmine R. Kirkwood, Jane Dickson, Marryat Stevens, Areti Manataki, Robert S. Lindsay, Deborah J. Wake, Dip (Med Ed) and Rebecca M. Reynolds in Journal of Diabetes Science and Technology
