# Supplementary material for: The User-Centered Design of a Clinical Dashboard and Patient-Facing App for Gestational Diabetes
Source: J Diabetes Sci Technol. 2024 Nov 29:19322968241301792. Online ahead of print. doi: 10.1177/19322968241301792 (PMC11607713; doi:10.1177/19322968241301792)
Supplement: sj-docx-1-dst-10.1177_19322968241301792 – Supplemental material for The User-Centered Design of a Clinical Dashboard and Patient-Facing App for Gestational Diabetes [file sj-docx-1-dst-10.1177_19322968241301792.docx]

# Supplementary material 1: Interview guides

## Health care professionals

Pre-interview

- Greet and introduction.
- Explain the interview - the interview will last around 30 minutes and the questions will involve four topics – about you and your experiences with providing care for gestational diabetes and the use of digital tools in the services you provide.
- Gain verbal consent for audio-recording the interview and remind participants that they do not have to answer any questions if they do not feel comfortable and can stop the interview at any time.

*Underlined questions are key questions asked in every interview.*

| **Research Question** | **Key Concepts** | **Example Interview Questions** |
| --- | --- | --- |
| What is current care like that health care professionals provide to women with gestational diabetes? | Current care  Improvements  Caregiving | Describe to me how you care for women with gestational diabetes.  How often will you see a patient with gestational diabetes during their pregnancy?  Do you provide a woman with gestational diabetes any education on their condition? If so, how do you provide this?  Do you recommend/do you patients uses any non-hospital resources for education or advice for women with gestational diabetes?  (e.g. eLearning course, websites)  What are the barriers and enablers for women to comply with recommended lifestyle changes or blood glucose monitoring/recording?  Do you think/feel there could be any improvements in the area of care you provide for women with gestational diabetes? What would these improvements be?  What system is in place for getting women who had gestational diabetes to return for their 12-week postpartum and yearly glucose screening? |
| What technology/digital tools and types of data are captured in the care of women with gestational diabetes? | Technology  Data type/capture  Current care | Explain to me the type of technology, if any, you use in the care you provide for women with gestational diabetes.  Do you suggest or recommend any technology or digital tools to your patients with gestational diabetes that are not provided by the hospital? If so, please explain to me what they are.  Does the technology used in gestational diabetes care you provide capture any form of data, if so, what does it capture? |
| What are the gestational diabetes health care professional’s acceptability of digital tools and computer decisions/support in gestational diabetes care? | Digital tool/technology  Acceptability | Explain to me how confident you would be if computer decision-making was part of the care pathway for gestational diabetes.  Would you be confident with a digital tool giving you or women with gestational diabetes automated advice or suggestions that were generated through machine learning? Such as highlighting women who have consistently had high blood glucose readings or giving women with gestational diabetes feedback on their glucose readings.  How helpful do you think a tool that predicts women’s pregnancy complications or outcomes would be? For example, predicting which women are more likely to need mediation or have a caesarean section.  What risks do you see with having a computer assistant with decisions in gestational diabetes care?  What benefits or limitations do you think there could be for both you and your patients for the use of digital tools or computer decisions? |
| What would healthcare professionals want from a digital tool that helps with gestational diabetes care and management? | Digital tool  Platform/interface  Feature | How would you like a digital tool to help you with the care and management of women with gestational diabetes?  If a digital tool were developed for healthcare professionals who care for gestational diabetes, what features would you want it to have?  How do you anticipate the support system could support HCPs in providing care?  How would you like to access this digital tool for healthcare professionals?  If a digital tool was developed for the women with gestational diabetes that you care for, what features would you want it to have for them?  How would you like your patients with gestational diabetes to access this digital tool? |

## Women with GDM

Pre-interview

- Greet and introduction.
- Explain the interview - the interview will last around 10/30 minutes and the questions will involve four topics about you and your experiences with gestational diabetes and the use of digital tools in the services you receive.
- Gain verbal consent for audio-recording the interview and remind participants that they do not have to answer any questions if they do not feel comfortable and can stop the interview at any time.

*Underlined questions are key questions asked in every interview.*

| **Research Question** | **Key Concepts** | **Example Interview Questions** |
| --- | --- | --- |
| What was the care like for women with gestational diabetes? | Current care  Improvements  Experience of GDM | Describe your experience with gestational diabetes.   - How was it taking BG to read regularly - How was going to extra appointments - How was changing diet or increasing exercise   What treatment plan are you on? (diet, metformin, insulin)  How is this treatment?  Explain to me how you managed your gestational diabetes.  How often did you have to go to the clinic or have appointments?  What was this like?  How did you find the education for gestational diabetes? Did you use only the hospital resources, or did you search for more information as well?  Was it/do you think/ do you feel easy or hard to remember everything you were told?  Was/do you think/do you feel the advice and education on diet tailored to your cultural or food preferences?  No? How could this be improved?  How was the adaption to lifestyle changes, and what were the barriers or enablers to these changes?  What were your main worries or concerns about having gestational diabetes and its possible problems, e.g. large baby, type 2 diabetes?  What did you like and not like about gestational diabetes care?  What improvements do you think there could be? /What would you change?  Postpartum only. How were you reminded about your 12-week postpartum OGTT? And did you go to that appointment? |
| What technology/digital tools and type of data is captured in the care of women with gestational diabetes? | Technology  Data type/capture  Current care | Did you use any technology for your gestational diabetes (or pregnancy) and if so, what were they?  Was this suggested or supplied by the hospital?  How easy or hard was it to use?  Did it capture any data? If so, what did it record?  Did you use any other technology to help you manage your gestational diabetes that wasn’t recommended by the hospital? Please explain to me what this was.  How did you find this, was it beneficial or not? |
| What are women with gestational diabetes' acceptability to digital tools or technology for gestational diabetes care? | Digital tools/technology  Acceptability | Do you think/feel that if there was a digital tool that had some form of automated advice or suggestion on gestational diabetes management you would find this helpful?  Such as giving instant feedback on your blood sugar reading letting you know if you’re in the right range or not and how to make the corrections if needed.  Explain to me how confident would you be if computer-based decision-making was used in your gestational diabetes care. For example, the computer could make the decision based on the blood sugar reading you’ve recently had that you should increase your physical activity.  What about computer assistance with medication?  Administrating?  Predicting if you were to need it?  How comfortable would you be with a computer predicting that you are more likely to have complications associated with GDM?  What complications do you think it would be helpful to predict? |
| What would women with gestational diabetes want from a digital tool that helped with gestational diabetes care and management? | Digital tool  Platform/interface  Features | What would you like a digital tool to help you within gestational diabetes care and management?  If a digital tool was made for gestational diabetes, how would you like to access it?  What would you want from a digital tool for gestational diabetes care? |
