# Supplementary material for: The User-Centered Design of a Clinical Dashboard and Patient-Facing App for Gestational Diabetes
Source: J Diabetes Sci Technol. 2024 Nov 29:19322968241301792. Online ahead of print. doi: 10.1177/19322968241301792 (PMC11607713; doi:10.1177/19322968241301792)
Supplement: sj-docx-2-dst-10.1177_19322968241301792 – Supplemental material for The User-Centered Design of a Clinical Dashboard and Patient-Facing App for Gestational Diabetes [file sj-docx-2-dst-10.1177_19322968241301792.docx]

Supplementary Material 2 – Feedback Sessions

# Feedback session guide

## Prototype Demonstration

Demonstrate both the clinical dashboard and patient app to the feedback group.

Dashboard (Figma prototype demonstration)

- Home page
- Home page icons
- Example patients, 7,3 and 1
- Message to patients
- Add new patients

Slides (PowerPoint)

- Example of medication prediction outputs

App (Figma prototype demonstration)

- Home page
- Home page, add notes and/or treatments
- Menu
- Request a call-back
- Menu – food log main page (including recipes and GDM community)
- Home -> education
- Medication
- Medication quiz

Slides (PowerPoint)

- Example of notifications for patients

## Discussion points/starting questions

| Key Concepts | Example Questions |
| --- | --- |
| Notification of off-target blood glucose | 1. What do you think about the dashboard notifying you of off-target patients? 2. What do you think of the in-app notifications and reminders for your patient if they are off-target? |
| Education, information and resources | 1. What do you think about the education resources for your patients are they [helpful, useful, appropriate]? |
| Usability | 1. Do you think this would increase/decrease workload? And why? 2. Could you see this being beneficial to your clinic and/or patients? And why? 3. Did the dashboard (and app for patients) seem easy or hard to use? And why? 4. Do you feel that the app is socially and culturally appropriate for your patient’s demographic? |
| Predictive modelling/risk stratification | 1. What do you think/feel about the risk stratification of patients through predicted medication needs? |
| Overall | 1. [Likes/dislikes], [Improvements/missing] of any section. 2. How do you see the dashboard and app being used in your clinics? 3. What do you think of patients being able to request a callback? 4. What do you think about being able to send patients a message? 5. What do you think of setting up/adding new patients? |

# Characteristics of feedback session participants

**Table 1 Characteristics of 31 participants in the three feedback sessions that evaluated the MyGDM prototype.**

|  | **Role** | **Number** |
| --- | --- | --- |
| Feeback session 1 | Clinical consultant and academic | 2 |
|  | Research midwife | 7 |
|  | Clinical research facilitators | 4 |
|  | PhD Students and clinical research fellows | 3 |
| Feeback session 2 | Consultant Obstetrician | 3 |
|  | Consultant Diabetologist | 4 |
|  | Diabetes Specialist Nurse | 4 |
| Feeback session 3 | Diabetic specialist midwife | 4 |

# Iterative changes from feedback sessions

**Table 2 Elements that were iteratively added to the MyGDM prototype from each feedback session (*SMBG: Self-monitoring blood glucose)***

| **Feedback session** | **Elements added to the MyGDM prototype** |
| --- | --- |
| 1 | Call back information:   - Specify who is going to be called. - Add office hours. - Explain what concerns the function should be used for. - Add a triage number for other concerns. |
|  | Add a technical support section. |
| 2 | Clinical dashboard sort/filter function.   - Add the ability to sort clinical dashboard: - Off-target SMBG most to least, and least to most. - Call back request only. - Warning only. - Alphabetically. |
|  | Visualization of patient’s information:   - Add visualization: - Line graph of SMBG readings. - Pie chart of on- and off-target SMBG. |
| 3 | Clinical dashboard sort/filter function:   - By clinical location. - By medication type. |
|  | Visualization of patient’s information:   - Format the patient’s SMBG in a logbook style, grouping readings by day. |
|  | Highlight which SMBG readings had been edited or added manually. |
