# Supplementary material for: The User-Centered Design of a Clinical Dashboard and Patient-Facing App for Gestational Diabetes
Source: J Diabetes Sci Technol. 2024 Nov 29:19322968241301792. Online ahead of print. doi: 10.1177/19322968241301792 (PMC11607713; doi:10.1177/19322968241301792)
Supplement: sj-docx-3-dst-10.1177_19322968241301792 – Supplemental material for The User-Centered Design of a Clinical Dashboard and Patient-Facing App for Gestational Diabetes [file sj-docx-3-dst-10.1177_19322968241301792.docx]

Supplementary Materials 3 – Questionnaire

# Questionnaire

Participants will be asked to watch a short video that demonstrates the MyGDM prototype app and then to complete the questionnaire.

**Questionnaire**

Welcome

Thank you for taking part in the study to assess the prototype MyGDM app.

In this study, we wish to find out what women with gestational diabetes first impressions are of the MyGDM app.

All the information we collect during the research will be kept confidential and there are strict laws which safeguard your privacy at every stage. However, NHS Lothian cannot guarantee the safety of your information when you are using your own devices (e.g., your own mobile or laptop).

Background

1. **Age**
   1. Under 16
   2. 16-20
   3. 21-25
   4. 26-30
   5. 31-35
   6. 36-40
   7. 41-45
   8. 46-50
   9. 50 +
   10. Prefer not to answer
2. **Ethnic identity**
   1. Asian/Asian British
      1. Indian
      2. Pakistani
      3. Bangladeshi
      4. Chinese
      5. Any other Asian background, please describe
   2. Black / African / Caribbean / Black British
      1. African
      2. Caribbean
      3. Any other Black / African / Caribbean background, please describe
   3. Mixed / Multiple ethnic groups
      1. White and Black Caribbean
      2. White and Black African
      3. White and Asian
      4. Any other Mixed / Multiple ethnic background, please describe
   4. White
      1. English / Welsh / Scottish / Northern Irish / British
      2. Irish
      3. Gypsy or Irish Traveller
      4. Any other White background, please describe
   5. Other:
      1. Please state
   6. Prefer not to say
3. **When were you diagnosed with gestational diabetes?**
   1. 0-7 days ago
   2. 8-14 days ago
   3. 15-30 days ago
   4. 1 to 2 months ago
   5. 3 to 6 months ago
   6. 7 to 9 months ago
4. **Do you have any other children?**
   1. Yes
   2. No
5. **Have you had gestational diabetes in a previous pregnancy?**
   1. Yes
   2. No
6. **What medication did/do you take for your gestational diabetes?**
   1. None
   2. Metformin
   3. Insulin
   4. Don’t know
   5. Prefer not to say
7. **Would you like to be updated with a short report once the study is completed?**
   1. Yes
   2. No

Questionnaire

1. **Do you think that the app presented here would have helped manage your gestational diabetes?** [select one]
   1. Yes, it would have helped manage my gestational diabetes.
   2. No, it would not have helped manage my gestational diabetes.
   3. Comments
2. **Could you see this app fitting in with your lifestyle to help manage your gestational diabetes?**
   1. **Yes**, it would have fitted in with my lifestyle,
   2. **No**, it would not have fitted in with my lifestyle.
   3. Comments
3. On a scale of 1 to 5, 1 being very unlikely and 5 being likely.

**How likely, if you had need, would you have used the ‘request a call’ feature?**

(very unlikely) 1 2 3 4 5 (very likely)

1. On a scale of 1 to 5, 1 being very unlikely and 5 being likely.

**How likely, would you have used the educational resources?**

(very unlikely) 1 2 3 4 5 (very likely)

1. On a scale of 1 to 5, 1 being very unlikely and 5 being likely.

**How likely, would you have used the course/quiz?**

(very unlikely) 1 2 3 4 5 (very likely)

1. **What do you like the most about the digital tool?** [short answer]
2. **What do you like the least about the digital tool?** [short answer]
3. **Is there anything that you expected the prototype type to have that you think is missing?**

[short answer]

1. **Do you have any other comments that you would like to add about this digital tool prototype?** [short answer]
   1. Yes

Please state:

- 1. No

Thank you!

Thank you for taking the time to complete the questionnaire.

If you have any further questions please contact Jazz Kirkwood, j.r.kirkwood@sms.ed.ac.uk

# Characteristics of questionnaire participants

**Table 1 Characteristics of 13 participants who took the questionnaire to evaluate the MyGDM prototype app**

| **Overview** | **Value** | **Number (percentage response, %)** |
| --- | --- | --- |
| Age (years) | 21-25 | 2 (15.4) |
|  | 26-30 | 2 (15.4) |
|  | 31-35 | 4 (30.8) |
|  | 36-40 | 4 (30.8) |
|  | 41-45 | 1 (7.7) |
| Ethnicity | Asian/Asian British | 2 (15.4) |
|  | Mixed / Multiple ethnic groups | 2 (15.4) |
|  | White | 9 (69.2) |
| Gestational diabetes diagnosis before taking the questionnaire | 8 to14 days ago | 1 (7.7) |
|  | 15 to 30 days ago | 2 (15.4) |
|  | 1 to 2 months ago | 2 (15.4) |
|  | 3 to 6 months ago | 3 (23.1) |
|  | 7 to 9 months ago | 5 (38.5) |
| Other children | Yes | 7 (53.9) |
|  | No | 6 (46.2) |
| Gestational diabetes in a previous pregnancy | Yes | 6 (46.2) |
|  | No | 7 (53.9) |
| Treatment for gestational diabetes at the time of the questionnaire | Diet | 5 (38.5) |
|  | Metformin | 5 (38.5) |
|  | Metformin and Insulin | 3 (23.1) |
